# Fractures of the proximal femur and hip osteoarthrosis – coincidence or coherence?

**DOI:** 10.1051/sicotj/2024027

**Published:** 2024-08-19

**Authors:** David Latz, Erik Schiffner, Christos Koukos, Falk Hilsmann, Joachim Windolf, Johannes Schneppendahl

**Affiliations:** 1 Department of Orthopedics and Trauma Surgery, Heinrich Heine University Hospital Duesseldorf Moorenstr. 5 40225 Duesseldorf Germany; 2 Sports Trauma and Pain Institute 54655 Thessaloniki Greece; 3 Department of Orthopedics and Trauma Surgery, Evangelisches Krankenhaus Mühlheim/Ruhr Wertgasse 30 45468 Müheim an der Ruhr Germany

**Keywords:** Hip, Osteoarthrosis, Fracture, Proximal femur, Intracapsular

## Abstract

*Purpose*: The current study aimed to investigate the correlation between the grade of radiographic hip osteoarthritis (OA) and the fracture pattern observed in fragility fractures of the proximal femur. The information may help in cases of occult hip fractures. *Methods*: In this retrospective study all 448 patients treated with fragility fractures of the proximal femur in the years 2014–2018 were included. Patients were allocated into two groups: Group I) intracapsular (femoral neck) fractures and Group II) extracapsular (pertrochanteric and subtrochanteric) femoral fractures. The radiographic grade of OA was determined according to Kellgren and Lawrence’s classification. One single observer examined all radiographs. *Results*: Patients’ age ranged between 52 and 104 years with a mean of 80.0 years. There was a significant difference in mean age between the two groups (76.9 years intracapsular vs. 83.1 years extracapsular fractures). A total of 250 (55.8%) fractures were intracapsular (femoral neck) and 198 (44.2%) were located extracapsular (pertrochanteric, subtrochanteric). A significant correlation between the degree of OA to fracture pattern was observed: Higher degrees of OA were related to extracapsular fractures and lower degrees of OA to intracapsular fractures. *Conclusion*: The results of this study support the hypothesis that hip osteoarthritis affects the fracture pattern in proximal femur fractures. More severe hip OA is associated with extracapsular fractures that can be treated surgically with lower complication rates compared to intracapsular fractures.

## Introduction

Considering society’s increasing life expectancy, fractures of the proximal femur have become a major health issue worldwide. In fact, this type of injury is the most expensive and debilitating among all osteoporotic fractures with an incidence predicted to at least double within the next decades [1, 2]. Epidemiologic studies were able to identify several risk factors, including age, sex, alcohol abuse, tobacco consumption, glucocorticoids, previous history of fracture, or low body mass index [2–4]. Despite numerous studies considering hip fractures as a homogenous group, two generic categories need to be distinguished: intracapsular (femoral neck) hip fractures and extracapsular (trochanteric) fracture localization. Intracapsular fractures are located medial to the attachment of the hip joint’s capsule to the proximal femur, whereas extracapsular fractures are found lateral to the capsule. If the fracture occurs below the trochanter region, the terminology “subtrochanteric femur fracture” is used. The distinction between intra- and extracapsular fractures is paramount for the management and prognosis of the injury. There is common agreement that the vast majority of hip fractures, whether it is trochanteric or femoral neck fractures, should undergo surgical treatment if the patient is stable enough to survive the intervention.

While the majority of extracapsular fractures are treated with internal fixation, there is controversy about whether to treat intracapsular hip fractures with internal fixation or (hemi-) arthroplasty [5, 6]. Approximately 95% of hip fractures in the elderly occur due to falls, in which the majority tumble sideways out of a standing height [7, 8]. Many studies have investigated the structural failures of the proximal femur leading to these fragility fractures caused by low-energy trauma. In this context, several authors described a decreased incidence of hip osteoarthritis (OA) in hip fracture patients compared to the general population [9, 10]. This implies a protective effect of hip OA on hip fractures. Other authors have described hip OA as protective only against intracapsular fractures, with unaffected rates of extracapsular fractures [11]. The authors suggested that this is based on an increased bone mineral density (BMD) in the region of the femoral neck with reduced BMD within the trochanter region in osteoarthritic hips [9, 12]. Previous studies varied greatly in methodology and their definition of hip OA. Some used patient self-report to evaluate OA [10, 13], while others applied radiographic definitions [9, 14]. Further, some authors did not differentiate between intra- and extracapsular fracture localizations [10]. Therefore, interpreting the available evidence is difficult.

An established patient cohort, also subject of other evaluations was used for the current study to investigate the correlation of radiographic OA and the fracture pattern observed in fragility fractures of the proximal femur.

## Methods

The local ethics committee approved this retrospective study of a patient cohort and was also evaluated in further studies, such as an investigation of the correlation of caput-collum-diaphyseal angle and fracture pattern. All patients with a fragility fracture of the proximal femur who were treated at one Level 1 Trauma Center between 01.01.2014 and 31.12.2018 were screened. Exclusion criteria were pathological hip fractures, advanced malignant diseases, and previous radio- or chemotherapy. Overall, 448 patients with hip fractures were included. Patients were allocated into two groups according to the documented diagnosis: Group I) intracapsular (femoral neck) fracture and Group II) extracapsular (pertrochanteric and subtrochanteric) femoral fracture. In all cases pre- and postoperative digital radiographs were available. All X-rays were obtained in a standard AP view of the pelvis with the same focal film distance. All radiographs were examined by one experienced observer. Based on the preoperative radiograph osteoartritis was graded according to Kellgren and Lawrence’s classification [15]. Therefore, the presence of osteophytes, subchondral sclerosis, periarticular ossicles, pseudo cystic areas, and an altered bone shape was assessed. Joint space narrowing was not enlisted as a criterion due to its tendency to be falsified through fracture hematoma, specifically in cases of intracapsular fractures. Furthermore, age, gender, height, and weight were obtained from the patient’s electronic file. Data was analyzed using Superior Performing Software System (SPSS), Version 22 for Windows (SPSS, Inc., Chicago, III). Arithmetic mean (AM) and standard deviation (SD) of age, height, weight, and degree of OA were calculated within the two cohorts. For statistical analysis, the Kruskal-Wallis non-parametric test followed by the post-hoc Dunn’s test and the chi-squared test was used to compare the grade of OA between the group of intra- versus the group of extracapsular fracture localization. Statistical significance was defined as a value of *p* < 0.05.

## Results

A total of 448 proximal femoral fractures were included. The age ranged between 52 and 104 years with a mean age of 80.0 years. There was a significant difference in mean age between the two groups with 76.9 years for intracapsular and 83.1 years for extracapsular fractures (*p* < 0.05). There was a significant difference in body height with an average of 169.6 cm for intracapsular fractures compared to 165.3 cm in the group of extracapsular fractures (*p* < 0.05). Also, a significant difference in body weight with an average of 70.2 kg for intracapsular fractures and 65.8 kg for extracapsular fracture patients was observed (*p* < 0.05). Details of the patient-specific data are displayed in Table 1.

**Table 1:** Patients characteristics, ± SD.

	Extracapsular	Intracapsular
*n*	198	250
Men	86	103
Women	112	147
Age (years)	83 (±11.2)	76 (±13.8)
High (cm)	165 (±7.93)	170 (±9.2)
Weight (kg)	66 (±14.1)	70 (±14.3)
BMI (kg/m^2^)	24(±4.1)	24 (±4.2)

A total of 250 (55.8%) fractures were intracapsular femoral neck fractures and 198 (44.2%) were located extracapsular as either pertrochanteric or subtrochanteric fractures. The number of subtrochanteric fractures was small, so these were grouped as extracapsular together with the pertrochanteric fractures (Table 2).

**Table 2:** Kellgren and Lawrence classification distribution, *n* = number of patients.

Group	Grade 1	Grade 2	Grade 3	Grade 4	Total
Extracapsular	17 (14.4%)	77 (43.5%)	82 (65.0%)	22 (81.5%)	198 (44%)
Intracapsular	101 (85.6%)	100 (56.5%)	44 (35.0%)	5 (18.5%)	250 (56%)
*n*	118 (p < 0.05)	177 (p < 0.05)	126 (p < 0.05)	27 (p < 0.05)	448

With regards to the hip OA degree, the following distribution pattern was observed: None of the patient’s OA was classified as grade 0. Overall 118 patients presented with grade 1 OA (26.3%), 177 patients with mild OA (39.5%), 126 patients with moderate changes (28.1%), and 27 patients with severe OA of the hip (6.0%). Analyzing the degree of OA according to the fracture pattern a significantly higher rate of intracapsular fractures was observed in the group of grade 1 and grade 2 OA. Patients with grade 1 OA suffered extracapsular fractures in only 14.4%, whereas 85.6% suffered intracapsular fractures. In grade 2 OA this difference was less obvious, but still significant with 43.5% extracapsular versus 56.5% intracapsular fractures.

In contrast, the groups of grade 3 and grade 4 hip OA presented significantly more extracapsular hip fractures. In grade 3 OA 65% extracapsular and 35% intracapsular fractures were observed. Patients with grade 4 OA suffered extracapsular fractures in 81.5% and with 18.5% intracapsular fractures are rare in severe OA (Table 2).

## Discussion

Despite multiple studies examining fragility fractures of the hip and the potential protective effect of OA, the majority of studies did not consider the degree of hip OA and its influence on the fracture pattern observed [16–19]. This study aimed to shed light on the question of whether the grade of OA of the hip correlates to the fracture pattern observed.

Presenting a mean age of 80.0 years this evaluation presents with demographics comparable to previous reports in which the mean age was 77.7 to 85.5 years [2, 7]. Aguado-Maestro et al. [16] evaluated the OA distribution according to Kellgren and Lawrence’s classification in a UK population. In this study, a relationship between the degree of OA and fracture pattern has been observed and patients with higher degrees of OA were more likely to suffer extracapsular fractures. The authors concluded that with an increasing grade of hip OA, the possibility of sustaining an extracapsular fracture increases. However, in the current study, the degree of OA was determined differently from Aguado-Maestro et al. [16]. In this context according to Kellgren and Lawrence’s classification no grade 0 hip OA has been observed and the majority of patients presented with significant OA. In comparison, Aguado-Maestro et al. [16] reported on a population in which 93.9% presented with minimal OA. This difference may be related to the definition of OA or the methodology used. In the present analysis the ratio of intracapsular to extracapsular was 1.3:1. This is comparable to previous reports [16, 17].

Similar to this study Calderazzi et al. [17] stated that osteoarthritis does not protect from proximal femoral fractures, but affects the location of the fracture of the proximal femur. The ratio of intra- and extracapsular fractures observed by Calderazzi et al. [17] was 1.7:1 however, OA of the hip led to increased rates of pertrochanteric fractures. Similar to this data the present study shows a correlation between higher degrees of OA to extracapsular fractures whereas lower degrees of OA are correlated with intracapsular fractures in a German population. A possible explanation for this relationship is the reduced range of motion (ROM) of the osteoarthritic hip. In a cadaveric study, Bings et al. [20] demonstrated that hips with higher ROM sustained intracapsular fractures whereas lower ROM led to extracapsular fractures. Since higher degrees of OA result in a decreased ROM this may substantiate the higher rate of extracapsular fractures in this population.

The impact of bone density on fracture patterns is discussed in the literature. Glowacki et al. advanced hip OA can be associated with a higher bone density at the femoral neck and trochanter but not intertrochanteric. This discrepancy at the femoral neck and intertrochanteric region may have an impact on fracture pattern [21]. Arokoski et al. suggest that hip OA is not significantly associated with a higher bone density in the femoral neck. However, the slightly increased bone density in the femoral neck in patients with hip OA may play a part in the pathogenesis of the fractures [22].

The influence of OA on fracture the pattern may be of interest in cases of occult proximal femur fractures. At our institution patients with traumatic hip pain and negative or equivocal radiographs receive CT scans to detect fractures of the proximal femur. However, Mandell et al. [23] reported for a small number of operatively treated proximal femoral fractures a sensitivity of CT scan of 60% (3/5) with a negative predictive value of 97%. Despite these accurate results for the CT scan, there may still be potential for grading OA in radiographs to detect the fracture pattern.

Other factors influencing fracture localization, such as the anatomical configuration of the proximal femur, have also been evaluated in the patient cohort used. Besides the shown grade of OA, a correlation of the column centre diaphysis angle has also been shown. This information may help to detect fractures. This is of great importance since the treatment of intra- and extracapsular fractures differs significantly. Extracapsular fractures are usually reduced and stabilized using implants such as a dynamic hip screw or intramedullary nailing [24]. In intracapsular fractures in elderly patients, the treatment significantly depends on patient factors, such as activity level, age comorbidities and fracture-dislocation. Besides reduction and fixation various arthroplasty options are used [25].

Numerous studies show lower post-operative mortality, perioperative blood loss, and surgery-related complications for the treatment of extracapsular fractures compared to arthroplasty in intracapsular fractures [26–28]. Since higher degrees of OA are associated with extracapsular fractures hip osteoarthritis indirectly protects patients from complications in the treatment of hip fractures.

The surgical goal of treatment in extracapsular fractures should be the achievement of a stable osteosynthesis that allows early full weight-bearing mobilisation of the patient [29]. However, in patients with ipsilateral hip osteoarthritis primary hip arthroplasty should be considered the primary treatment modality to reduce the likelihood of a secondary procedure [30]. Mäkinen et al. recommend prosthetic replacement for unstable extracapsular fractures as an alternative primary treatment option [30]. However, a clear indication of primary hip arthroplasty in the treatment of extracapsular fractures does not exist [31]. Furthermore, primary hip arthroplasty eliminates the possibility of malunion, cut-out of the hip screw, avascular necrosis of the femoral head and a secondary procedure [31]. However, primary arthroplasty is a technically challenging procedure. Fichmann et al. [32] compared complications and clinical outcomes of primary hip arthroplasty to osteosynthesis for the treatment of extracapsular fractures. The authors observed primary hip arthroplasty offers a lower re-operation rate in the treatment of extracapsular fractures compared to osteosynthesis. Further studies are needed to analyze the optimal surgical treatment for patients with ipsilateral hip osteoarthritis and extracapsular fractures.

This study has some limitations, such as its retrospective design No information was available for BMD at the proximal femoral area.

## Conclusion

The results support the assumption that higher degrees of OA are correlated with intracapsular fractures. This information may help to predict fracture patterns in fragility fractures of the proximal femur.

## Data Availability

The data that support the findings of this study are available from the corresponding author upon reasonable request.
